# Quality Management Systems implementation in public medical laboratories; A sustainable approach to health system strengthening in Lagos State, Nigeria

**DOI:** 10.1371/journal.pone.0319409

**Published:** 2025-06-02

**Authors:** Tolulope Adaran, Olanrewaju Jenrola, Feyisayo Jegede, Oluwafunmilayo Ojo, Adebayo Bakare, Olakunle Omiyale, Olaniyi Felix Sanni, Omoh A Aliu, Babatunde O Owolabi

**Affiliations:** 1 Grant Management Unit, Lagos State Ministry of Health, Lagos, Nigeria; 2 Laboratory Services Department, Directorate of Disease Control, Lagos State Ministry of Health, Lagos, Nigeria; 3 Department of Life Science, Bayero University Kano, Kano, Nigeria; 4 Audit Unit, Strengthening Laboratory Management Towards Accreditation, Lagos, Nigeria; 5 Research and Development Department, Fescosof Data Solutions, Ogun State, Nigeria; 6 State Tuberculosis Control Program, Directorate of Disease Control, Lagos State Ministry of Health, Lagos, Nigeria; 7 Department of Health Sciences, Canadore College, Ontario, Canada; Nigerian Institute of Medical Research, NIGERIA

## Abstract

**Background:**

The effectiveness of Quality Management Systems (QMS) in public medical laboratories is crucial to ensure quality and reliable testing outcomes for quality healthcare. This research aims to achieve a minimum of 2 Stars WHO-AFRO rating at the external audit of ten public medical Laboratories within twelve months of intervention using improved documentation and institutionalization of robust QMS.

**Method:**

A quasi-experimental design was used to assess QMS interventions in ten of 28 public secondary medical laboratories in Lagos State. These facilities were randomly selected using non-probability measures over 12 months from November 2022 to October 2023. The study measured resource allocation, conducted staff training for capacity building, and provided mentoring support. External audits were performed using the WHO-AFRO SLIPTA 2015 checklist, with a grading system from 0 to 5 stars. The data collected included baseline and post-intervention scores, analyzed using descriptive statistics and baseline compared with follow-up audit performance.

**Result:**

The 12-month implementation of laboratory QMS in ten Lagos State’s public secondary health facilities revealed substantial progress. Nine Medical laboratories in the study had a baseline WHO-AFRO rating of 0 Star, while General Hospital Ikorodu had a baseline rating of 1 Star. Sixty percent of the medical laboratories demonstrated commendable QMS improvement and achieved 3 Stars WHO-AFRO rating each, while twenty percent of the medical laboratories attained 2-Stars each. However, the remaining twenty percent of the health facilities achieved minimal improvements, securing 1 Star WHO-AFRO rating each.

**Conclusion:**

Overall, Eighty percent of the medical laboratories showed progress in QMS implementation in Lagos State. The study reveals that a Government-led QMS implementation drives a more sustainable culture of quality in medical laboratories and the twelve-month measure indicates the possibility to extend the QMS interventions to the remaining eighteen public medical laboratories in Lagos State.

## Background

Medical Laboratory services is a critical component of the health care system generating data that help physicians make informed decisions. Errors from the Medical laboratory services could negatively impact patient health outcomes. Globally, Quality Management Systems (QMS) are crucial building blocks interconnected to minimize errors in medical laboratory services. Medical laboratories faced significant challenges to ensure test results’ quality, reliability, accuracy, and timeliness standards [1]. Implementing a QMS can help laboratories overcome these challenges by implementing with fidelity in the application of the twelve quality system essentials as composite building blocks that help fulfill predetermined quality objectives [2].

Lagos State, Nigeria’s vibrant metropolis ensures quality healthcare hinges on robust public laboratories. These laboratories serve as the frontline sentinels, providing accurate diagnoses, monitoring disease outbreaks, and supporting clinical decision-making [3]. However, the effectiveness of these crucial institutions can be hampered by inadequate quality management systems, leading to unreliable results, compromised patient care, and, ultimately, weakened health systems [1]. Implementing robust QMS in public medical laboratories is not merely a technical endeavour but a transformative force in strengthening healthcare infrastructure. Ojo *et al.* [4] state, “Quality management is at the heart of an effective health system and is essential for ensuring safe, effective, and patient-centred care.” Embedded within this statement lies the fundamental purpose of this research: to explore the institutionalization of QMS in public medical laboratories across Lagos State, proposing a sustainable and measurable approach that fosters health system strengthening over a period.

As Kleinman & Dougherty [5] emphasize, “Quality improvement in health care is not an event and not a destination but a journey,” this research embarks on that journey, striving to equip public medical laboratories in Lagos State with the tools and systems necessary to navigate the path towards healthcare excellence. In a measure to ensure an efficiently strengthened laboratory system, the Lagos State Ministry of Health (LSMOH), through funding support from the Deutsche Gesellschaft für Internationale Zusammenarbeit (GIZ) grant, put strategies in place to ensure the implementation of a sustainable laboratory quality management system for continuous improvement procedures and overall guidance to achieve a minimum of 2-STARS WHO-AFRO rating using SLIPTA checklist after twelve months. The QMS program includes a set of policies, procedures, and practices that monitor/control all steps of the medical laboratory service cycle that can affect laboratory results ranging from pre-analytic, analytic, and post-analytical phases [6].

In 2020, the Global Fund supported the mapping of medical laboratories, which identified 26 secondary public laboratories with poor QMS implementation, hence the need to channel resources to improve the quality of medical laboratory service. The general findings from these 26 Lagos State-owned secondary facilities include sparing knowledge of QMS, leading to a lack of commitment to implementation in the entire medical laboratory service. In contrast, some levels of implementation were identified only in the HIV-disease-specific donor-funded laboratories in the facilities. This significant gap has not factored in integrated service delivery for a holistic approach to quality medical laboratory services. This fragmented approach (support for only disease-specific labs) reveals the knowledge gap in the implementation and sustainability of systems through policy development that will ensure the commitment of facility management to quality system essentials [7]. Hence, there is a need for capacity building, continuous monitoring of compliance with standards and measures of compliance to ensure sustainability. Ensuring knowledge sharing among these facilities to institutionalize the culture of QMS for a strengthened medical laboratory service [8].

The major aim of this study is to measure the impact of the various QMS interventions geared towards building the capacity of the Laboratory staff in compliance with ISO 15189;2012 using the WHO-AFRO SLIPTA checklist for rating.

### Objectives

1. To assess the level of QMS implementation in the 10 supported health facilities.
2. To analyze the impact of the various QMS interventions in the state.
3. To provide recommendations to stakeholders, resource mobilization and for policy brief.

## Methodology

### Study design

This study adopted a Quasi-experimental design to assess the impact of QMS interventions in ten selected public secondary laboratories in Lagos State, Nigeria. The interventions aimed to strengthen laboratory systems and achieve a minimum 2-STARS WHO-AFRO rating within 12 months.

### Study area

The study was conducted across ten public secondary medical laboratories in Lagos State, Nigeria. These laboratories were strategically selected based on their need for QMS improvement and minimal infrastructural upgrade requirements. Lagos State being a state in southwestern Nigeria that borders Ogun State to the East and North, it is both the most populous and smallest in area of the 36 states in Nigeria. Lagos State is known for its vibrant culture, bustling markets, and significant economic activities. The state is a key culture, education, and transportation hub for Nigeria and Sub-Saharan Africa. Lagos has recorded medical breakthroughs in combating Ebola and COVID-19 outbreaks. The state also has the highest literacy rate in Nigeria [9].

### Population, sample frame and respondents

Lagos State has a total of twenty local government areas with 28 secondary health facilities. All of the 28 secondary health facilities were considered target population for this intervention, while 10 out of the secondary health facilities fit into the sample frame. The respondents comprised medical laboratory staff, laboratory managers, quality officers, safety officers and other personnel directly involved in laboratory operations across the ten selected facilities. The 10 supported facilities are:

1. General Hospital Lagos
2. General Hospital Ikorodu
3. General Hospital Isolo
4. General Hospital Gbagada
5. General Hospital Onikan
6. General Hospital Randle, Surulere
7. General Hospital Epe
8. General Hospital Badagry
9. Mainland Hospital Yaba
10. Lagos Island Maternity Hospital

### Justification for the selection of 10 secondary health facilities

The ten facilities that met the inclusion criteria such as the availability of good laboratory infrastructure (building/location) within a hospital setting with minimal or no need for renovations, high patient in-flow, with a minimum of five technical staff to ensure adequate dedication to QMS implementation and concurrence of the hospital management to ensure commitment to QMS implementation were selected. The participating facilities received equal support during this research.

### Ethical consideration

Ethical considerations are a crucial aspect of research that ensures the protection of human rights, dignity, and safety, as well as the integrity of scientific data [10].

Ethical considerations included adherence to standard protocols for conducting audits and training sessions. All interventions and data collection processes were conducted with the full approval by the Health Research and Ethics Committee of Lagos State University Teaching Hospital (LASUTH-HREC) for Lagos State Government following ethical guidelines.

### Data collection, training of personnel, resources allocation, and support/ external audit

The data were collected from the scores obtained at audits conducted at the facilities using the SLIPTA checklist 2015 version tool, these facilities’ scores were obtained from the Resilient and Sustainable Systems for Health (RSSH) of the Directorate of Healthcare Planning, Research and Statistics (DHPRS) of the Lagos State Ministry of Health. Data collection took place from August 20th to September 20th 2024.

The review of the data covered the interventions conducted at implementation which are:

#### Training programs.

Various training sessions were conducted to enhance the capacity of laboratory staff in QMS implementation, Biosafety, biosecurity, ISO 15189: 2012 compliance, and internal audit processes. These sessions were both residential and non-residential, accommodating different aspects of QMS using different training approaches.

#### Resource allocation and support.

The provision of stationery and deployment of Laboratory Information Management Systems (LIMS) at the selected laboratories were part of the support mechanisms. There was the visit of selected Quality Officers from other disease-specific implementing laboratories to conduct a hand-holding session for the development of a draft of Quality Manual at the 10-supported facilities. Each facility was visited by a 2-man team to guide the focal officers in the facility to develop the draft documents. Furthermore, mentoring visits by QMS mentors were undertaken to assist in on-site hand-holding processes and document development.

#### External audits.

The interventions were audited using the WHO-AFRO SLIPTA checklist 2015, employing a team of two auditors over three days to conduct external audits in October 2023. This checklist contains 12 sections which assess Baseline scores were recorded in November 2022 to assess the progress and impact of the various interventions, in the following sections, Documents and Records, Management Reviews, Organization and Personnel, Client Management and Customer Service, Equipment, Evaluation and Audits, Purchasing and Inventory, Process Control, Information Management, Identification of Non-Conformities, Corrective and Preventive Actions, Occurrence/Incident Management and Process Improvement, and Facilities and Safety.

### Data analysis

Descriptive statistics test was used to analyze the quantitative data from the WHO-AFRO SLIPTA checklist 2015 version for each facility at the external audits. The descriptive statistics presented outcomes as frequency tables, percentages, and bar charts. The statistical test performed a Pre-post analysis, comparing the change in SLIPTA baseline scores before and after the interventions, and WHO-AFRO ratings were tabulated, data cleaning was performed and assessed. This is to measure the impact of the interventions on laboratory ratings (Table 1).

**Table 1:** SLIPTA audit ratings.

No Stars	1 Star	2 Stars	3 Stars	4 Stars	5 Stars
(0 – 150 pts) * < 55%*	(151–177pts) *55 – 64%*	(178–205 pts) *65 – 74%*	(206–232 pts) *75 – 84%*	(233–260 pts) *85 – 94%*	(261–275 pts) ≥*95%*

The above table is the standard Audit rating for the measure of QMS using the SLIPTA Checklist 2015 edition to measure the compliance of the facilities with ISO 15189;2012 edition.

## Results

The results from the external audit utilizing the WHO SLIPTA Checklist underscore significant strides made within the 12-month implementation period across the laboratories in Lagos State as shown in Table 2. Remarkably, several facilities demonstrated commendable advancements, with Lagos Island Maternity Hospital, Mainland Hospital Yaba, General Hospital Lagos, General Hospital Gbagada, General Hospital Onikan and General Hospital Ikorodu achieving a noteworthy 3 STARS each. These high-performing institutions showcased substantial adherence to quality management systems, marked by impressive score increments from their baseline assessments. However, while displaying moderate improvements, some facilities, such as General Hospital Isolo, and General Hospital Randle attained 2 STARS, signifying the need for further enhancements to reach higher rating tiers. Conversely, General Hospital Badagry and General Hospital Epe exhibited minimal improvements, securing 1 STAR each, highlighting the imperative for additional support and interventions to meet the required standards. This collective progress emphasizes the dedication to implementing and sustaining quality management systems, albeit underscoring the necessity for continued efforts and support, particularly for facilities requiring additional assistance, to ensure hospital management’s commitment to comprehensive quality enhancements across all public laboratories in Lagos State.

**Table 2:** Facility compliance evaluation outcome using WHO SLIPTA checklist 2015 (Nov 2022 - Oct 2023) with two auditors over three days.

SN	Facility Name	Baseline Audit – Nov 2022		External Audit - Oct 2023	
		% Score	Star Rating	% Score	Star Rating
1	Lagos Island Maternity Hospital (LIMH)	49.0%	**0 Star**	82.4%	**3 Stars**
2	Mainland Hospital Yaba (MHY)	52.7%	**0 Star**	80.7%	**3 Stars**
3	General Hospital Lagos	48.4%	**0 Star**	76.4%	**3 Stars**
4	General Hospital Isolo	46.5%	**0 Star**	70.5%	**2 Stars**
5	General Hospital Gbagada	38.5%	**0 Star**	84.5%	**3 Stars**
6	General Hospital Ikorodu	55.0%	**1 Star**	78.6%	**3 Stars**
7	General Hospital Badagry	49.0%	**0 Star**	56.0%	**1 Star**
8	General Hospital Epe	43.0%	**0 Star**	59.0%	**1 Star**
9	General Hospital Onikan	43.3%	**0 Star**	81.2%	**3 Stars**
10	General Hospital Randle	52.9%	**0 Star**	66.4%	**2 Stars**

**Fig 1:**
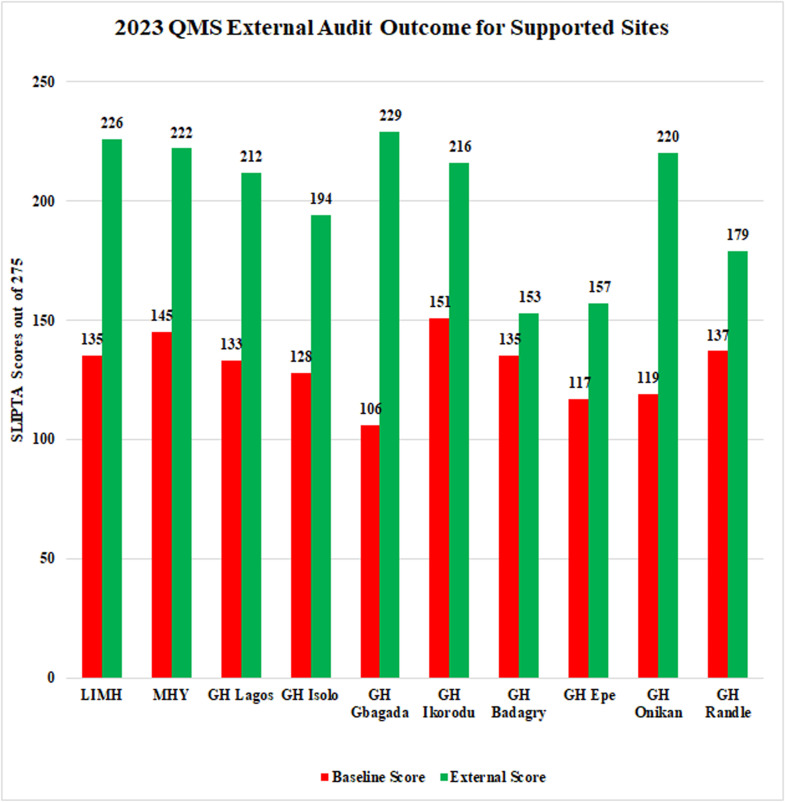
Baseline and External Audit Performance across 10 Medical Laboratories in Lagos.

Fig 1 shows the 2023 QMS External Audit Outcome for Supported Sites, with SLIPTA scores out of 275. GH Gbagada achieved the highest external audit score of 229 while GH Epe recorded the highest baseline score of 157.

The external assessment reviewed the 12 sections of the audit checklist and the breakdown of scores per health facility is in the table 3:

**Table 3:** 10 Medical laboratories final audit performance score across the 12 QMS sections assessed.

Section	Total points	LIMH	MHV	GH Lagos	GH Isolo	GH Gbagada	GH Ikorodu	GH Badagary	GH Epe	GH Onikan	GH Randle
SECTION 1: Documents and Records	28	28	15	26	25	23	18	14	20	19	20
SECTION 2: Management Reviews	14	14	6	14	11	10	14	0	1	4	10
Section 3: Organisation and personnel	22	17	22	22	19	20	22	13	13	22	14
SECTION 4: Client management and customer service	101	10	9	10	5	10	10	6	8	9	5
SECTION 5: Equipment	26	22	27	26	24	26	19	18	19	28	15
SECTION 6: Evaluation and Audits	15	11	15	15	11	15	10	11	11	11	11
SECTION 7: Purchasing and Inventory	24	21	22	15	15	24	20	14	20	23	22
SECTION 8: Process control	32	28	30	26	25	25	29	18	17	29	23
SECTION 9: Information management	19	15	17	10	16	11	16	11	12	16	14
SECTION 10: Identification of Non-conformities, Corrective and Preventive Actions	19	17	11	9	7	15	11	14	11	12	11
SECTION 11: Occurance/Incident Management and process improvement	12	10	10	6	6	11	10	2	4	10	6
SECTION 12: Facilities and safety	43	33	38	33	30	39	37	32	21	37	28
**TOTAL SCORE**	**275** **(100%)**	**226**	**222**	**212**	**194**	**229**	**216**	**153**	**157**	**220**	**179**

The summary of findings of Non conformities across 10 medcial laboratories in lagos and recommedsations was assessed in Table 4.

**Table 4:** Summary of non-conformities across 10 medical laboratories in Lagos.

	Sections	Summary of Findings: Non-Conforming Events	Recommendations
**1**	**Documents & Records**	No evideence of review of records appropriately and actions taken such as on patients’ results, temperature charts, etc. Documents of external origin were not identified as such and not included in the masterlist. Unavailablity of appropriate shelves for documents. Obsolete documents are still found in circulation as at the time audit.	Ensure necessary documentations are completed when reviews are conducted Provide a stamp to identify the documents of external origin. Provide well secured and labelled shelves for documents. Ensure that all obsolete controlled documents are dated and marked as obsolete. At least one copy of an obsolete controlled document is retained for a specified time period or in accordance with applicable specified requirements.
**2**	**Management Reviews**	There was no evidence of review of quality objectives and the quality policy for appropriateness and continuous improvement during management review meeting.	The laboratory should ensure that the quality objectives and policy are reviewed annualy for improvement to meet the demands of the customers.
**3**	**Organization & Personnel**	No evidence seen that the QM reports to management at which decisions relating to quality are made on the Organogram. No Root cause analysis evidence for resolution of non conforming works and customer complaints seen. At the time of this audit, no documented evidence that medical surveillance has been conducted.	Provide evidence that the QM reports to management at which decisions relating to quality are made on the Organogram. Provide Root cause analysis evidence for resolution of non conforming works and customer complaints. Lab to conduct medical surviellance for its staff and document in their personnel folders.
**4**	**Client Management & Customer Service**	No specific complaints procedure sighted in the client handbook as at the time of audit. No adequate evidence seen for communication of delays in the turnaround time or service interruptions to clients.	The laboratory should provide its clients with a handbook that outlines the laboratory’s hours of operation, available tests, procedure for complaints,specimen collection instructions, packaging and shipping directions, and expected turnaround times. Provide adequate evidence for communication of delays in the turnaround time or service interruptions to clients
**5**	**Equipment**	At the time of this audit, there were no record of installation verification. No documented estimate of Measurement of Uncertainty	The lab should perform a retrospective verification on installed equipment, including the back-up equipment. Determine the estimate of MoU
**6**	**Evaluation and Audit**	Policy exist but no process for implementation of potential pitfalls seen. No evidence of non-conformance report for Internal Audit Report and RCA for NCs seen	Provide evidence of process for implementation of potential pitfalls. Provide evidence of non-conformance report for Internal Audit Report and RCA for NCs.
**7**	**Purchasing & Inventory**	No evidence of budgetary projection sighted at the time of audit. No record of inspection of reagents received.	The laboratory shoul ensure that budgetry projection should bbe made at the beginning of the year to prevent distruption of service. Provide record of inspection of reagents received
**8**	**Process Control**	Samples for Comparison or proficiency testing are not tracked at the reception showing that they are treated with specialty. There is no evidence as at the time of audit that the lab evaluate and review their referral labs.	The laboratory should ensure that samples for proficiency testing and interlaboratory comparison are handled, analyzed, reviewed and report results in a manner similar to regular patient testing. The laboratory should establish a system for selection and evaluation of referral labs. and consultants.
**9**	**Information Management**	There was no detailed procedure for Laboratory information sysytem as at the time of audit. No validation or review of results before release.	The lab should establish detailed procedure for the use of LIS in the lab to include how regular services would be carried out and how root cause anaylssis and corrective actions will be carried out. All results must be reviewed/validated before release.
**10**	**Identification of Non-Conformities, Corrective and Preventive action**	The determination of extent of the non-conformity is not well defined in the SOP. Root causes are not fully determined in line with standard.	The lab should establish a detailed procedure for determing the extent of non-conformity and steps to take when a requester is to be recalled for a repeat of test. The lab should establish a process to ensure that all non-conformities are carried out in line with the standard and documented.
**11**	**Occurrence/Incident Management & Process Improvement**	There was no evidence to show actions taken, checked and monitored to determine the effectiveness of Quality indicators (QI). No evidence that graphical tools are used to communicate quality findings and no communication with the management on QIP	The lab should create an action plan to monitor the QI stating the objectives, methodology, interpretation, limits, action plan and duration of measurement for each QI. Graphical tools should be used to communicate quality findings and communicate with management on Quality Improvement Projects.
**12**	**Facilities and Biosafety**	Access control is not fully implemented. Guideline on vaccination was not sighted in the manual as at the time of audit. No evidence of safety risk assessment of the laboratory.	The lab should ensure that lab is strictly restricted for unauthorized personnel. Guidelines on vaccination should be included in the biosafety manual. Risk assessment should be conducted to determine biosecurity risk and mitigation plan should be put in place.

## Discussion

The primary purpose of the medical laboratory is to generate data that are accurate, precise, and useful for clinical decision-making by the physicians, contributing to improve patient outcomes and optimal management of patients. This can not be achieved without a well-functioning QMS within the laboratory. QMS is therefore essential for efficiency, ensuring quality results for patient care. Evaluating the laboratory through assessments is an effective method to ascertain its accuracy, reliability, and adherence to Good Clinical Laboratory Practices GCLP). The roles played by a medical laboratory are pivotal, as it promptly delivers quality, accurate, and reliable laboratory data to meet the requirements of physicians engaged in medical practice and patient health maintenance expected health outcomes [11]. Therefore, it is imperative to minimize the potential for errors and uphold the highest achievable quality standards in testing and results [12].

The findings of this study underscore the critical importance of implementing sustainable QMS in public medical laboratories within Lagos State. The study revealed substantial progress. Almost all the 10 medical laboratories in the study had a baseline rating of 0 stars, except General Hospital Ikorodu with a baseline rating of 1 star. Remarkably, a good proportion of the medical laboratories (60%) demonstrated commendable QMS improvement in Lagos Island Maternity Hospital, Mainland Hospital Yaba, General Hospital Lagos, General Hospital Gbagada, and General Hospital Ikorodu achieving an important milestone of 3 Stars each WHO-AFRO Rating. However, moderate improvements were observed in 20% of the medical laboratories (General Hospital Isolo and General Hospital Randle) that attained a 2-Stars WHO-AFRO Rating. The remaining 20% (General Hospital Badagry and General Hospital Epe) achieved minimal improvements, securing 1 Star each.

The comparison between the baseline scores recorded in November 2022 and the final scores obtained in October 2023 showcases the substantial strides made in the QMS implementation. Facilities that initially lacked stars due to lower baseline scores now boast significantly higher scores, signifying a positive shift in their adherence to quality standards – ISO 15189;2012. Notably, General Hospital Gbagada displayed a remarkable improvement, soaring from a baseline score of 106 (0 Star) to an impressive 229, attaining a 3-star rating within the study period.

Similar studies have been conducted in other countries, such as Uganda, where implementing a quality management system in public laboratories led to significant improvements in the quality of laboratory services [13]. Another study conducted in Nigeria showed improved WHO/AFRO ratings after the audit [14]. The study also found that implementing QMS in a public hospital improved patient outcomes and increased patient satisfaction [14]. These studies suggest implementation of QMS in public medical laboratories is a sustainable approach to strengthening health systems.

In contrast, a study in Oyo state, Nigeria, assessed the capacity of public secondary facility-based medical laboratories to conduct diagnostic tests for selected epidemic-prone diseases. Capacity was assessed on a 100-point scale in which scores were rated low. Diagnostic testing capacity for bacterial meningitis, cholera, and measles was “low” in all the laboratories. The reasons reported for laboratories not conducting diagnostic tests for the selected diseases included inadequate instruments, unavailable reagents, and clinicians’ failure to request those diagnostic tests [15]. Findings from another study in Ghana in which a QMS audit was performed, the laboratory audit showed an overall weak laboratory QMS (zero star rated) based on the WHO/AFRO laboratory strengthening checklist and rating [12].

The possible reasons for improvement in the laboratory quality management system in this study are a result of the Lagos State government’s driven approach to management commitment and evidenced sustainability for public medical laboratory facilities, unlike what is currently experienced in the country where implementation is largely driven by donor partners in disease-specific laboratories. Resource mobilization using government counterpart financing to build the capacity of laboratory staff is critical to support other donor funds and continuous monitoring of activities to ensure the Medical Directors of these facilities account for the various health system strengthening interventions.

The various trainings and rounds of mentorship provided by the SLMTA Nigeria team may have improved the skills and knowledge of the laboratory staff in understanding the standard, leading to better development of documents, adherence to standard operating procedures (SOPs) and overall quality management practices [16]. Also, regular internal and external audits may have helped identify areas for improvement in a laboratory’s quality management system. Addressing audit findings can drive continuous improvement [16]. The effectiveness of Quality Management Systems (QMS) significantly relies on the services provided by auditors and assessors who play a crucial role in maintaining and sustaining standards. In 2017, the CDC-Nigeria laboratory program, in partnership with the ASLM team, conducted ISO15189 training. This initiative aimed to enhance the capabilities of 24 auditors and SLMTA personnel within the country, enabling them to better assist laboratories in achieving higher service quality and adherence to international standards. Consequently, there is a pressing need to formulate comprehensive in-country auditing guidelines tailored explicitly for medical laboratories in Nigeria [16].

Another reason for the improvement in QMS of the laboratory system in this study could be attributed to various forms of technical support, including infrastructure upgrades, capacity building, improvement projects, follow-up visits, mentoring, and advocacy by the Lagos State Ministry Health team, the measurable quality improvement is likely a direct outcome of these efforts. Furthermore, the commitment demonstrated by the technical staff at the facilities, coupled with a certain degree of ownership and support from facility management, has also played a significant role in the program’s achievements [14].

Despite the advancements made in QMS within public secondary medical laboratories as indicated in this study, there is an ongoing need for improvement. Implementing QMS services is crucial to ensure the reliability and reproducibility of results. Raising awareness about the significance of laboratories adopting QMS and maintaining such a culture should be given high priority. It is also recommended to encourage more laboratories to participate in the WHO/AFRO SLMTA/SLIPTA program, seek experienced mentors, and pursue accreditation. These efforts will contribute to a well-organized and consistent workflow, uphold standards, deliver quality and competent services, and enhance customer satisfaction.

### Limitations of the study

Limitations of this study stem from several factors. Firstly, a short intervention period of twelve months might not entirely reflect the long-term sustainability or overall impact of implemented quality management systems. Secondly, the absence of detailed discussion on resource constraints and external factors, like policy changes or staff turnover, raises concerns about potential unaccounted influences on the outcomes. Reliance on a single assessment tool, the WHO-AFRO SLIPTA checklist limits the depth of analysis. Thirdly, insufficient exploration of ethical considerations and human resource dynamics further constrains the study’s comprehensive understanding.

## Conclusion

The GIZ Grant target for the measure of quality management systems was to attain a minimum of 2 stars within twelve months of implementation. The outcome of the ten laboratories revealed that six health facilities achieved 3 Stars, 2 of the facilities attained 2 stars, while two facilities showed a level of improvement to attain 1- Star. Hence, demonstrates a significant possibility to strengthen the culture of QMS for quality service delivery.

## Supporting information

S1 Data QMS Lab analysis. (XLSX)

## References

[1] World Health Organization. Laboratory quality management system handbook. 2011:1–247.

[2] WHO Africa. WHO Guide for the Stepwise Laboratory Improvement Process Towards Accreditation in the African Region. AFRO Libr Cat Data [Internet]. 2015;2–31. Available from: http://www.afro.who.int/en/clusters-a-programmes/hss/blood-safety-laboratories-a-health-technology/blt-highlights/3859-who-guide-for-the-stepwise-laboratory-improvement-process-towards-accreditation-in-the-african-region-with-checklist.html

[3] Uchejeso M, Maduka K, Bassey I, Maduabuchi K. Improving quality and cost diminution in modern healthcare delivery: The role of the medical laboratory scientists in Nigeria. International Journal of Business Management and Inventory. 2019;8(3):8–19.

[4] Ojo OA, Asogba SO, Adewole TO, Amodu MO, Odeyemi O. Laboratory quality management system and its impact on quality healthcare delivery in Nigerian hospitals. Int J Heal Res Rev. 2020;4(4):32–8.

[5] Kleinman LC, Dougherty D. Assessing quality improvement in health care: theory for practice. Pediatrics. 2013;131 Suppl 1:S110-9. doi: 10.1542/peds.2012-1427n 23457146

[6] Rodma P, Silva P. SEA-HLM-403 Distribution: General Guidelines for implementation of quality standards for health laboratories; 2009

[7] (NACA). NA for the C of A. HIV/AIDS Control in Nigeria. Role Qual Manag Syst Dis Lab. 2020.

[8] WHO. Nigerian laboratory receives WHO full accreditation for fight against measles and rubella. 2023; Available from: https://www.afro.who.int/countries/nigeria/news/nigerian-laboratory-receives-who-full-accreditation-fight-against-measles-and-rubella

[9] Obeng-Odoom F. The State of African Cities 2010: Governance, inequality and urban land markets. Cities. 2013;31:425–9. doi: 10.1016/j.cities.2012.07.007

[10] Anjankar AJ, Mohite PM, Waghmode A, Patond S, Ninave S. A Critical Appraisal of Ethical issues in E-Learning. Indian Journal of Forensic Medicine & Toxicology. 2021;15(3):78–81. doi: 10.37506/ijfmt.v15i3.15283

[11] Yokota H, Yatomi Y. Future roles of clinical laboratories and clinical laboratory technologists in university hospitals. Rinsho Byori. 2013;61(8):686–91. 24218765

[12] Tanko R, Aninagyei E, Baffour AG, Gbadago F, Addae G, Ameme D, et al. Assessment of Clinical laboratory using WHO AFRO-SLIPTA Quality Standards in a Referral Hospital laboratory in Ghana. 2021;1–13.

[13] Hage J, Valadez J, Nkolo C. Institutionalizing and Sustaining the Lot Quality Assurance Methodology in Uganda. 2014;

[14] Mbah H, Ojo E, Ameh J, Musuluma H, Negedu-Momoh OR, Jegede F, et al. Piloting laboratory quality system management in six health facilities in Nigeria. PLoS One. 2014;9(12):e116185. doi: 10.1371/journal.pone.0116185 25542022 PMC4277469

[15] Bankole OT, Ajayi IO. Assessment of laboratory capacity of public secondary health facilities in performing assay of selected epidemic-prone diseases in Oyo State, Nigeria. Diagn Microbiol Infect Dis. 2019;95(2):191–4. doi: 10.1016/j.diagmicrobio.2019.05.016 31296359

[16] Nwaokorie FO, Ojo EA. Overview of the implementation of quality management system in Nigerian medical laboratories. Univ Lagos J Basic Med Sci. 2018;6(1 & 2):20–9.

